# Can we more precisely classify exposure to antenatal depression and anxiety in multivariable prediction models of pregnancy and birth outcomes: a population-based cohort study

**DOI:** 10.1186/s12888-023-05284-9

**Published:** 2023-11-03

**Authors:** Grace A. Thiele, Deirdre M. Ryan, Tim F. Oberlander, Gillian E. Hanley

**Affiliations:** 1 Department of Gynaecology and Obstetrics, University of British Columbia (UBC), 828 West 10th Ave, Vancouver, BC V5Z 1M9 Canada; 2 Departments of Psychiatry, University of British Columbia (UBC), F605-4500 Oak St, Shaughnessy building, BC Women’s Hospital and Health Centre, Vancouver, BC V5Z 4H4 Canada; 3 Department of Pediatrics, University of British Columbia (UBC), 938 W 28th Ave, Vancouver, BC V5Z 4H4 Canada; 4 Department of Gynaecology and Obstetrics, University of British Columbia, Vancouver General Hospital Research Pavilion, 590-828 West 10th Ave, Vancouver, BC V5Z 1M9 Canada

**Keywords:** Perinatal mental health, Depression, Anxiety, Administrative data, Postpartum depression, Preterm birth

## Abstract

**Background:**

Depression and anxiety are highly prevalent within the perinatal period and have been associated with myriad adverse pregnancy and birth outcomes. In this study, we sought to investigate whether population-based data can be used to build complex, longitudinal mental health histories that improve our ability to predict adverse pregnancy and birth outcomes.

**Methods:**

Using population-based, administrative datasets, we examined individual-level mental health services use of all birth parents who delivered a live infant in British Columbia, Canada between April 1, 2000, and December 31, 2013, and who were registered with the provincial Medical Services Plan for over 100 days per year from 10-years preconception to 1-year postpartum. We operationalized variables to proxy severity, persistence, and frequency of depression/anxiety from preconception through pregnancy, then constructed predictive regression models for postpartum depression/anxiety and preterm birth.

**Results:**

Predictive modeling of postpartum depression/anxiety and preterm birth revealed better predictions and stronger performance with inclusion of a more detailed preconception mental health history. Incorporating dichotomous indicators for depression/anxiety across preconception markedly improved predictive power and model fit. Our detailed measures of mental health service use predicted postpartum depression/anxiety much better than preterm birth. Variables characterizing use of outpatient psychiatry care and outpatient visit frequency within the first five years preconception were most useful in predicting postpartum depression/anxiety and preterm birth, respectively.

**Conclusion:**

We report a feasible method for developing and applying more nuanced definitions of depression/anxiety within population-based data. By accounting for differing profiles of mental health treatment, mental health history, and current mental health, we can better control for severity of underlying conditions and thus better understand more complex associations between antenatal mental health and adverse outcomes.

**Supplementary Information:**

The online version contains supplementary material available at 10.1186/s12888-023-05284-9.

## Background

Depression and anxiety affect up to 18.4% and 20.7% of pregnant and postpartum people, respectively [1, 2], and research has suggested that they increase risk for adverse outcomes, ranging from pre-term delivery [3] to postpartum depression [4]. Prior history of depression and anxiety has been demonstrated as a strong predictor of perinatal (both antenatal and postnatal) mood disorder [5]. In one study, nearly half of those experiencing antenatal depression had a prior history of major depressive disorder [6].

Evidence from non-perinatal populations suggests that depression and anxiety are dynamic, varying in severity and propensity to remit [7]. Within pregnancy, studies report distinct, symptom-based trajectories that describe diverse experiences of perinatal depression [8]. These trajectories have been shown to be differentially associated with outcomes such as preterm birth [9], and gestational age [10]. Despite an advancing understanding of the heterogeneity of depression/anxiety, most population-based studies operationalize a dichotomous definition of perinatal depression/anxiety based on presence of a diagnostic code during a single period [11–14]. While these codes are validated in identifying psychiatric conditions [15], this approach misses an opportunity to capture different experiences of depression/anxiety that may be differentially associated with outcomes of interest, thus better controlling for the underlying condition.

Herein, we aim to determine whether longitudinal data on mental health services use could be leveraged to better differentiate between distinct phenotypes of depression/anxiety. We sought to operationalize variables that describe different aspects of care from 10-years preconception through pregnancy based on the presence of depression/anxiety diagnostic codes, including: any use of health services, frequency of outpatient visits, inpatient hospitalization, any psychiatry care, and persistence. We then examined how well these variables predicted two outcomes that have been previously associated with antenatal and/or preconception depression/anxiety, one with a more direct connection (postpartum depression or anxiety [PPD]) and one with a more indirect connection (preterm birth [PTB]).

## Materials and methods

In this retrospective cohort study, we examined data from all births in British Columbia (BC), Canada between April 1, 2000, and December 31, 2013. Birth parents were followed from 10-years preconception to one-year postpartum. Individual-level data from the following administrative datasets were accessed and linked through Population Data (PopData) BC: BC Perinatal Data Registry [16], BC Vital Events and Statistics: Births [17], Central Demographics File [18], Discharge Abstracts Database [19], and the Medical Services Plan (MSP) Payment Information File [20].

### Ethics approval

was granted by the University of British Columbia Behavioural Research Ethics Board and data steward approval was obtained from relevant data stewards for use of deidentified administrative data. Both approvals include a waiver of informed consent from participants. All inferences, opinions, and conclusions drawn are those of the authors and do not reflect opinions or policies of the Data Stewards.

### Study cohort

The BC Perinatal Data Registry (BCPDR) is a validated delivery-based resource for information on nearly 100% of births in BC [21]. It includes detailed data on obstetric history, current pregnancy conditions, antenatal care, and birth outcomes. These data were used to identify all births within the study period. We excluded non-singleton births and performed all analysis at the level of the pregnancy. To ensure completeness of preconception mental health data, we restricted our cohort to those registered with MSP for > 100 days per year from 10-years preconception to one-year postpartum. We excluded individuals with incomplete demographic information, and pregnancies without final gestational age (GA). Given our focus on depression/anxiety, we excluded birth parents with diagnostic codes for bipolar disorder, mania, schizophrenia, and psychosis.

### Measures

**Fig. 1:**
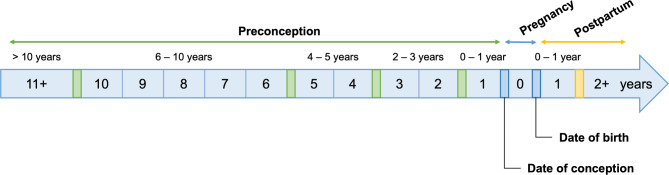
Representative timeline for periods of interest within constructed mental health histories. Preconception periods included: 0–1 year, 2–3 years, 4–5 years, 6–10 years, and > 10 years preconception. Curves were generated using RStudio.

*Time periods of interest.* The BCPDR estimates final GA using earliest ultrasound, where available—in the absence of ultrasound dating, GA is constructed using the birth parent’s last menstrual period (LMP). If missing, clinical examination of the newborn is used. We approximated date of conception (DOC) by subtracting GA from the infant’s date of birth (DOB) and subtracting two weeks. From DOC, we created five preconception periods (Fig. 1) and defined the antenatal period as time between DOC and the child’s DOB.

*Measures of depression/anxiety.* Depression and anxiety were treated as a composite exposure and outcome as they frequently co-occur among perinatal and non-perinatal populations [22, 23]; however, we ran a sensitivity analysis to assess the validity of this construct. Both depression and anxiety are often treated using antidepressants like selective serotonin-reuptake inhibitors, possess overlapping risk factors, and have similar neurological profiles [24–28]. There is also a BC specific diagnostic code that does not distinguish between depression or anxiety and represents either condition as 50B within the *International Classification of Disease, Ninth Revision, Clinical Modification* (ICD-9-CM). Characteristics of health service use has been used previously as a way of describing and controlling for birthing parent mental health [14, 29]. We used diagnostic codes from physician visit (ICD-9-CM) and hospital data (ICD-10-CM) to identify all inpatient and outpatient visits with a diagnostic code for depression/anxiety during periods of interest (Supplemental Table 1). From these codes, we built dichotomous variables for each period to indicate any health service use depression/anxiety. Health service use for depression/anxiety was further characterized by indicators for hospitalization, outpatient psychiatry care, and frequency of outpatient visits (low, medium, or high). Diagnostic codes were also used to exclude prespecified mental illnesses (Supplemental Table 2).

Persistence of preconception depression/anxiety was categorized into four groups: no prior history, episodic, chronic-discontinuous, and chronic-continuous. No prior history included incident cases of depression/anxiety occurring antenatally. Episodic illness described individuals with relevant codes in a single preconception period. Chronic illness included individuals treated for depression/anxiety during ≥2 periods and was subdivided based on treatment continuity. Chronic-discontinuous illness described cases with ≥4 years between two consecutive treatment periods. Chronic-continuous illness represented cases with ≤4 years between periods of treatment.

### Outcomes

A birth parent was determined to have PPD if they were treated in an inpatient or outpatient setting for depression or anxiety within one year from the child’s DOB. Any delivery prior to 37 weeks GA was designated as preterm.

### Covariates

Sociodemographic covariates included birth parent and coparent age, presence of a coparent, neighborhood-based income quintile, marital status, and number of living children. Pregnancy characteristics included birth parent smoking status during pregnancy, history of PTB, parity, pre-existing diabetes, gestational diabetes mellitus (GDM), pregnancy-induced and non-pregnancy-induced hypertension (PIH and non-PIH), and intrauterine growth restriction (IUGR). Prenatal care and delivery characteristics included year of birth, number of prenatal visits, antenatal hospitalization, use of induction, mode of delivery (vaginal vs. non-vaginal), and presence of a midwife. Finally, neonatal characteristics included: infant sex, GA, birth weight, and admission to the neonatal intensive care unit (NICU).

### Statistical analysis

Given the estimated prevalence of postpartum depression (8.7%) and preterm birth (9.7%) in BC as well as the maximum number of predictors we might include in our final models, we determined that we would need a minimum sample size of 3,500 pregnancies [30, 31]. Our inclusion criteria ensured high levels of completeness within our data, therefore we did not employ any imputation methods for missing data. If mental health data had been missing from a given time period, it would have indicated that a person was either not yet living or no longer living in the province, which would have required that all data for that time period would need to be imputed. Variables were compared between those with and without antenatal depression/anxiety using standardized mean differences (SMD), with a value of 0.1 indicating a clinically meaningful difference [32]. Our cohort was then divided randomly into training (70%) and validation (30%) datasets, which were compared using an identical descriptive analysis.

*Model building.* Using our training set, we constructed logistic regression models for each exposure group (depression, anxiety, and depression or anxiety) to predict the log likelihood of PPD or PTB. For each outcome, we built a **Base Model** that included our binary indicator of antenatal depression/anxiety. We then incorporated depression/anxiety indicators for each preconception period, working backward to build a more longitudinal mental health history (**Binary Models**). Our detailed measures (outpatient visit frequency, hospitalizations, and psychiatrist visits) were added systematically to determine the relative contribution of each parameter to model fit and predictive power (**Full Models**). Our persistence measure required data up to five-years preconception and was added to models according to years of data included.

We determined a parsimonious **Final Model** according to variable importance. We first assessed the effect of incorporating information from each preconception period, then assessed relative contribution of each mental health measure to model strength. Models were compared using degree of correlation (R^2^) and receiver operating characteristic (ROC) curves (C-statistic). We implemented a DeLong test to determine whether subsequent models resulted in statistically significant improvements in the C-statistic [33].

Final models were adjusted by socio-demographic factors (**Adjusted Model 1**), then pregnancy and neonatal characteristics (**Adjusted Model 2)**. Covariates were added in a stepwise fashion to determine if they improved model fit. Variables that significantly improved model fit were kept in the model. For both outcomes, we controlled for birth parent age category, marital status, number of living children, smoking status during current pregnancy, number of prenatal visits, antenatal hospitalization, and presence of a midwife. Our PPD adjustment also included PTB, and admission to the NICU, while our PTB adjustment included number of neighbourhood-based income quintile, history of PTB, pre-existing diabetes, GDM, PIH, non-PIH, IUGR, and neonatal sex.

*Model validation and performance.* We evaluated performance of our base model, final unadjusted model, and both adjusted models. ROC curves were constructed from the training cohort to determine a threshold probability that maximized model sensitivity and specificity and could classify predicted log likelihood of an outcome within the validation cohort. We assessed model performance using sensitivity, specificity, positive predictive value (PPV), negative predictive value (NPV), and balanced accuracy (mean of sensitivity and specificity). All statistical analysis was performed using RStudio statistical software (RRID: SCR_000432).

*Sensitivity analyses.* In order to validate the use of a composite variable to describe depression/anxiety, binary and full models were built using three classes of depression/anxiety diagnostic categories: depression, anxiety, and depression or anxiety (50B code). We compared C-statistics and R^2^ values across these models to determine whether differentiating between depression, anxiety, and the combined 50B code (depression or anxiety) had a meaningful impact on our results.

## Results

### Characteristics of the study population

There were 582,459 recorded births in BC between April 1, 2000, to December 31, 2013. Of these, we excluded all non-singleton births (N = 17,789), all birth parents not registered with MSP for at least 100 days per year from 10-years preconception to one-year postpartum (N = 491,346), all births without GA or demographic data (N = 1,028), and all birth parents with prespecified mental health conditions other than depression/anxiety (N = 2,335). Our final cohort consisted of 69,961 births to 63,461 birth parents.

Characteristics of included (N = 69,961) and excluded (N = 512,498) births are summarized in Supplemental Table 3. Within the final cohort, 13,013 (18.6%) had at least one diagnostic code for depression/anxiety during pregnancy (Table 1). Compared to birth parents without any relevant codes, birth parents with at least one diagnostic code for depression/anxiety were less likely be married (59.9% vs. 67.1%; SMD = 0.156) and less likely to have a co-parent (94.1% vs. 96.7%; SMD = 0.123). They were more likely to smoke during the current pregnancy (11.5% vs. 8.5%; SMD = 0.140), more likely to be hospitalized during pregnancy (12.5% vs. 8.8%; SMD = 0.120), and less likely to have a midwife (13.3% vs. 19.4%; SMD = 0.167). PPD was much more likely among those with depression/anxiety during pregnancy (45.1% vs. 16.7%; SMD = 0.647), whereas PTB was not meaningfully different (9.6% vs. 8.4%; SMD = 0.040). Mental health characteristics are compared between those with and without a diagnosis of antenatal depression or anxiety in Supplemental Table 4. Mental health characteristics among those with antenatal depression or anxiety are further described according to diagnostic category (depression, anxiety, and depression or anxiety [50B code]) in Supplemental Table 5. Descriptive analysis comparing the training (N = 48,973) and validation (N = 20,988) datasets revealed no meaningful differences in the distribution of any characteristics of interest (Supplemental Table 6).

**Table 1:** Comparison of characteristics between birthing parents with and without intrapartum depression or anxiety. A standardized difference of 0.1 or greater was deemed significant and designated with a (**)

	Intrapartum depression or anxiety		
	No N = 56,948	Yes N = 13,013	Standardized difference
**Birthing parent sociodemographic factors**			
Birthing parent age group, N (%)			0.063
< 20 years	1875 (3.3)	464 (3.6)	
20–24 years	7990 (14.0)	1817 (14.0)	
25–29 years	16,098 (28.3)	3541 (27.2)	
30–34 years	18,084 (31.8)	3948 (30.3)	
35–39 years	10,355 (18.2)	2537 (19.5)	
≥ 40 years	2546 (4.5)	706 (5.4)	
Neighborhood income quintile, N (%)			0.031
1	11,351 (19.9)	2657 (20.4)	
2	11,761 (20.7)	2679 (20.6)	
3	11,895 (20.9)	2793 (21.5)	
4	12,527 (22.0)	2870 (22.1)	
5	9414 (16.5)	2014 (15.5)	
Marital status, N (%)			0.156**
Divorced	931 (1.6)	298 (2.3)	
Married	38,223 (67.1)	7794 (59.9)	
Never married	13,610 (23.9)	3784 (29.1)	
Other	3201 (5.6)	808 (6.2)	
Single	983 (1.7)	329 (2.5)	
Co-parent status			
Co-parent listed, N (%)	55,043 (96.7)	12,242 (94.1)	0.123**
Co-parent age (years), Mean (SD)	33.2 (6.3)	33.5 (6.6)	0.051
Number of living children, N (%)			0.094
0	25,687 (45.1)	6467 (49.7)	
1	20,824 (36.6)	4278 (32.9)	
2	7037 (12.4)	1524 (11.7)	
3	2186 (3.8)	490 (3.8)	
4 or more	1214 (2.1)	254 (2.0)	
**Pregnancy characteristics and risk factors**			
Year of birth, Mean (SD)	2011.9 (0.9)	2011.9 (0.9)	0.002
Smoking status during pregnancy, N (%)			0.140**
No history of smoking	46,193 (81.1)	9812 (75.4)	
Continued during pregnancy	4835 (8.5)	1496 (11.5)	
Discontinued during pregnancy	5920 (10.4)	1705 (13.1)	
History of premature birth, N (%)	2401 (4.2)	586 (4.5)	0.014
Nulliparous, N (%)	25,298 (44.4)	6360 (48.9)	0.089
Diabetes, N (%)			
Preexisting	409 (0.7)	100 (0.8)	0.006
Gestational	5023 (8.8)	1471 (11.3)	0.083
Hypertension, N (%)			
Pregnancy-induced	3069 (5.4)	781 (6.0)	0.026
Other	1985 (3.5)	499 (3.8)	0.019
Prenatal care, N (%)			
≥10 prenatal visits	19,550 (34.3)	5046 (38.8)	0.092
Prior hospital admissions	4999 (8.8)	1621 (12.5)	0.120**
IUGR, N (%)	861 (1.5)	251 (1.9)	0.032
Nature of labour, N (%)			
Vaginal delivery	40,067 (70.4)	8718 (67.0)	0.073
Induced labour	11,707 (20.6)	2921 (22.4)	0.073
Midwifery care	11,051 (19.4)	1726 (13.3)	0.167**
**Postpartum and neonatal characteristics**			
Infant sex, N (%)	27,804 (48.8)	6296 (48.4)	0.009
Gestational age (weeks), mean (SD)	38.6 (1.9)	38.5 (1.9)	0.070
Size at birth			
Birth weight (g), mean (SD)	3437.0 (546.0)	3412.7 (550.5)	0.044
Small for gestational age*, N (%)	4704 (8.3)	1111 (8.5)	0.010
Large for gestational age*, N (%)	6288 (11.0)	1475 (11.3)	0.009
Admission to neonatal intensive care unit, N (%)	1086 (1.9)	295 (2.3)	0.025
Preterm birth, N (%)	4803 (8.4)	1245 (9.6)	0.040
Postpartum depression or anxiety, N (%)	9483 (16.7)	5869 (45.1)	0.647**
Depressive disorder(s)	4473 (7.9)	3191 (24.5)	0.465**
Anxiety disorder(s)	3607 (6.3)	2456 (18.9)	0.385**
Depression or anxiety (BC specific ICD-9 50B code)	3419 (6.0)	2352 (18.1)	0.377**

* Indicators of small for gestational age and large for gestational age were determined using birth weight deciles within strata based on final gestational age and infant sex

### Predictive model building

**Fig. 2:**
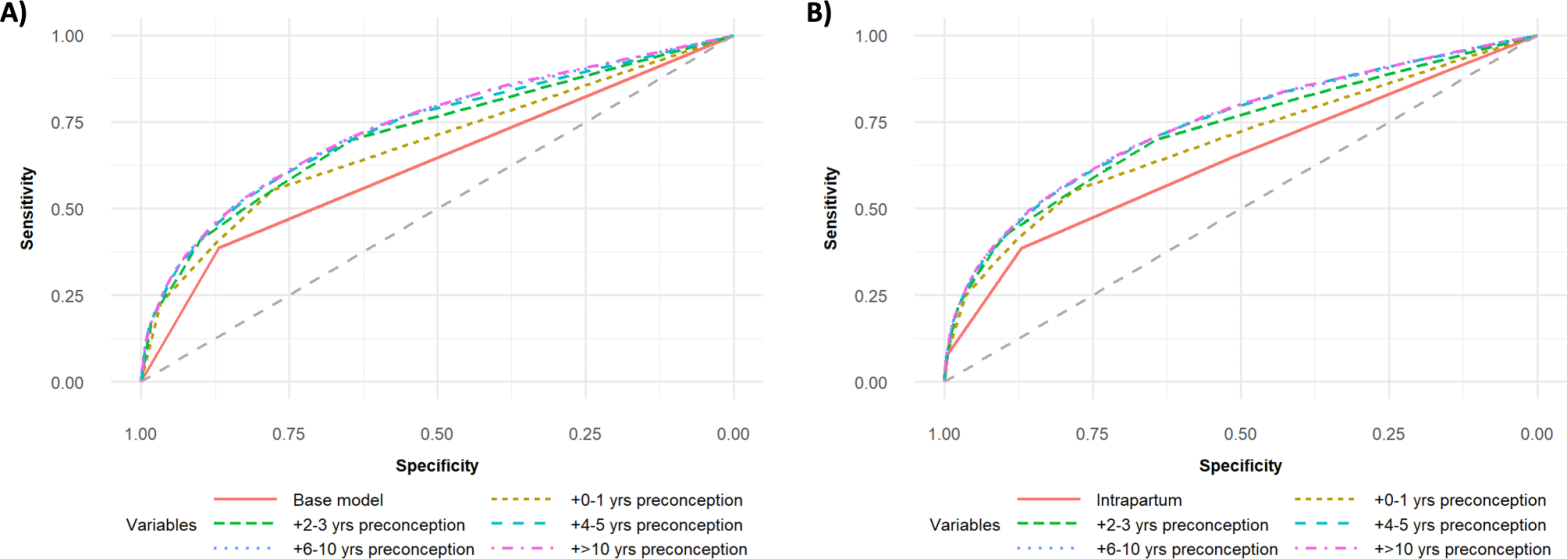
Receiver operator characteristic (ROC) curves for postpartum depression/anxiety (PPD) predictive models. Models were constructed using **(A) binary set** and **(B) full set of variables** describing mental health history. Variables from each preconception period were added in a stepwise fashion to the intrapartum base model, and each model is represented using a distinct color and line type. The expected ROC curve for predictions due to chance is represented using a dashed grey line. Curves were generated using RStudio

*Postpartum depression.* Introduction of binary depression/anxiety variables for each preconception period (**Binary Models**) resulted in a substantial increase in area under the ROC curve (AUROC) (Fig. 2A). Addition of data from the first-year preconception produced the greatest increase in AUROC (0.6268 vs. 0.6842, p < 0.001)and R^2^ (0.0953 vs. 0.1610). While sensitivity and specificity improved with the inclusion of each preconception period, the strength of this improvement attenuated as we moved further back in time (Fig. 2A). We observed a similar trend in predictive power and fit of our model, with modest changes in C-statistics (0.7283 vs. 0.7349, p < 0.001) and R^2^ values (0.2019 vs. 0.2068) after incorporating data from 6 to 10 years preconception.

Addition of our detailed mental health measures (**Full Models**)—frequency of outpatient health service use, hospitalization, use of psychiatric care, and depression/anxiety persistence—increased AUROC, but to a lesser degree (Fig. 2B). The data suggest the first five years of preconception mental health history are most predictive of PPD, and that use of psychiatric services and depression/anxiety persistence had the greatest impact on model strength. Our **Final Model** included all mental health measures within pregnancy, depression/anxiety and psychiatric services use indicators for the first five years preconception, and the depression/anxiety persistence measure.

**Fig. 3:**
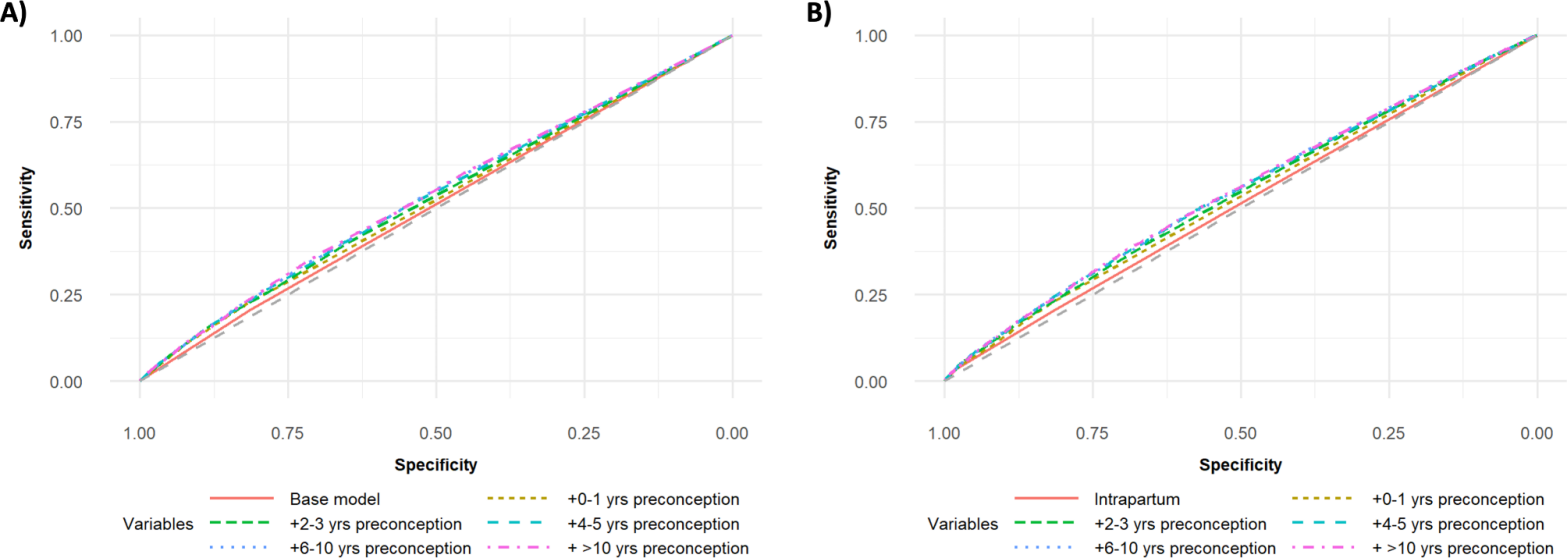
Receiver operator characteristic (ROC) curves for preterm birth (PTB) predictive models. Models were constructed using **(A) binary set** and **(B) full set of variables** describing mental health history. Variables from each preconception period were added in a stepwise fashion to the intrapartum base model, and each model is represented using a distinct color and line type. The expected ROC curve for predictions due to chance is represented using a dashed grey line. Curves were generated using RStudio.

*Preterm birth.* Overall association between PTB and antenatal depression/anxiety was weak, demonstrated by Fig. 3A. Inclusion of depression/anxiety indicators (**Binary Models**) increased the AUROC (0.5123 [95% CI: 0.5059–0.5186] vs. 0.5451 [95% CI: 0.5357–0.5542]), but all ROC curves were only slightly above the ROC for a random classifier (AUROC = 0.5). Improvement in model sensitivity, specificity, and AUROC attenuated with the inclusion of earlier preconception data. Models built using detailed mental health measures had slightly higher predictive power and fit compared to binary models from the same preconception period (Fig. 3B). Addition of a depression/anxiety indicator for 0–1 year (**Binary Model 2**) produced the largest increase in C-statistic (0.5123 vs. 0.5250, p < 0.001) and R^2^ value (0.0007 vs. 0.0027). Overall, mental health history from 0 to 5 years preconception was most predictive of PTB. With respect to individual variables, frequency of outpatient visits and depression/anxiety persistence appeared most predictive. All mental health measures for pregnancy were included in our **Final Model**, with binary depression/anxiety and frequency of outpatient service variables included for the first five years preconception, as well as the depression/anxiety persistence measure. See Supplemental Tables 7 & 8 for full summary of model C-statistics and R^2^ values.

### Model validation and performance

**Fig. 4:**
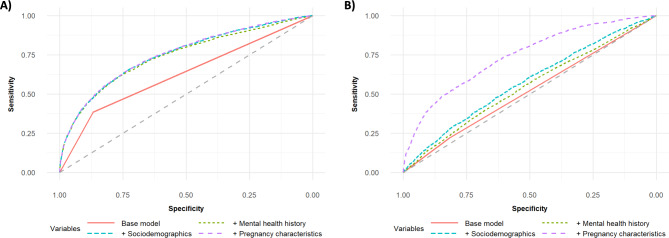
Receiver operator characteristic (ROC) curves for **(A) postpartum depression/anxiety ***and ***(B) preterm birth ***adjusted models*. Variables related to mental health history were selected according to resultant increase in concordance statistic (C-statistic). Other predictors (socio-demographics and pregnancy characteristics) were selected *a priori* and/or based on ability to significantly improve model fit. Each model is represented with a distinct color and line type. The expected ROC curve for predictions due to chance is represented using a dashed grey line. Curves were generated using RStudio

*Postpartum depression.* Measures of PPD model performance are summarized in Table 2, and ROC curves are provided in Fig. 4A. Adjustment for mental health history (**Final Model**), sociodemographic factors (**Adjusted Model 1**) and pregnancy or neonatal characteristics (**Adjusted Model 2**) all increased AUROC and R^2^ from the **Base Model**. Balanced accuracy improved with each adjustment, reaching a final value of 0.6819 (**Adjusted Model 2**). Introduction of mental health measures had the largest effect on model performance, significantly improving the C-statistic (0.6268 vs. 0.7348, p < 0.001), reducing distance between sensitivity and specificity by 69.9% (0.4922 vs. 0.1481), and improving balanced accuracy by 9.6% (0.6231 vs. 0.6829). A full summary of our final **Adjusted Model 2** is provided in Supplemental Table 11.

**Table 2:** Performance of predictive logistic regression models for postpartum depression using test dataset

Performance metric	Base model	Final model	Adjusted Model 1	Adjusted Model 2
	Intrapartum depression or anxiety (Y/N)	Partial history of mental health for intrapartum and up to 5 years preconception	**Final model** + birth parent age category + marital status + smoking status + number of living children	**Adjusted Model 1** + midwifery care + NICU admission + premature birth + antenatal hospitalization + over 10 prenatal visits
R^2^	0.0953	0.2122	0.2179	0.2212
C-statistic	0.6268	0.7348	0.7431	0.7460
Threshold probability	0.3074	0.2115	0.2070	0.2153
Sensitivity	0.3770	0.5922	0.6206	0.6078
Specificity	0.8692	0.7639	0.7411	0.7559
Balanced accuracy	0.6231	0.6781	0.6809	0.6819
PPV	0.4531	0.4188	0.4078	0.4170
NPV	0.8293	0.8670	0.8718	0.8703

***Abbreviations***: coefficient of determination (R^2^), concordance statistic (C-statistic), positive predictive value (PPV), negative predictive value (NPV), neonatal intensive care unit (NICU)

*Preterm birth.* Measures of PTB model performance are summarized in Table 3, and ROC curves are provided in Fig. 4B. Mental health history (**Final Model**), sociodemographic factors (**Adjusted Model 1**), and pregnancy or neonatal characteristics (**Adjusted Model 2**) each improved model AUROC, R^2^, balanced accuracy, PPV, and NPV. Addition of each set of predictors augmented model AUROC and reduced the distance between sensitivity and specificity. Performance was most improved with the addition of pregnancy or neonatal characteristics. Between **Adjusted Model 1** and **Adjusted Model 2**, C-statistic improved significantly (0.5650 vs. 0.7428, p < 0.001), balanced accuracy increased by 24.0% (0.5361 vs. 0.6647), PPV increased by 53.4% (0.1032 vs. 0.1583), and NPV increased by 3.5% (0.9223 vs. 0.9544). A full summary of our final **Adjusted Model 2** is provided in Supplemental Table 12.

**Table 3:** Performance of predictive logistic regression models for preterm birth using test dataset

Performance metric	Base model	Final model	Adjusted Model 1	Adjusted Model 2
	Intrapartum depression or anxiety (Y/N)	Partial history of mental health for intrapartum and up to 5 years preconception	**Final model** + birth parent age category + income quintile + marital status + smoking status + number of living children	**Adjusted Model 1** + history of premature birth + pre-existing diabetes + GDM + PIH + non-PIH + IUGR + midwifery care + prior antenatal hospitalization + over 10 antenatal visits + sex of infant
R^2^	0.0007	0.0059	0.0115	0.1535
C-statistic	0.5123	0.5476	0.5650	0.7428
Threshold probability	0.0906	0.0831	0.0897	0.0776
Sensitivity	0.1996	0.4776	0.3997	0.6611
Specificity	0.8156	0.5681	0.6725	0.6683
Balanced accuracy	0.5076	0.5229	0.5361	0.6647
PPV	0.0926	0.0945	0.1032	0.1583
NPV	0.9153	0.9202	0.9223	0.9544

***Abbreviations***: coefficient of determination (R^2^), concordance statistic (C-statistic), positive predictive value (PPV), negative predictive value (NPV), gestational diabetes mellitus (GDM), pregnancy-induced hypertension (PIH), non-pregnancy-induced hypertension (non-PIH), intrauterine growth restriction (IUGR)

### Sensitivity analysis

Ultimately, model performance did not meaningfully change when we stratified diagnoses into the three distinct groups of depression, anxiety, and depression or anxiety (BC specific ICD-9 50B). The largest difference in a C-statistic (0.0308) was observed in **Binary Models** for PPD that incorporated up to 10 years (**Model 5**) and over 10 years (**Model 6**) of preconception mental health history data. Similarly, the maximum difference in R^2^ (0.0658) was noted among **Full Models** for PPD incorporating over 10 years (**Model 6**) of preconception mental health history data. Most C-statistics and R^2^ values changed less than 0.02 and 0.06 respectively. Supplemental Tables 9 & 10 provide a full summary of the difference in C-statistic and R^2^ values between models built with each depression or anxiety diagnostic category.

## Discussion

In this population-level cohort study, we explored whether longitudinal health services data could be used to better describe individual-level depression/anxiety and thus better predict the likelihood of two distinct outcomes: PPD and PTB. We determined that dichotomous indicators of mental health across preconception improved predictive power and model fit. As expected, detailed measures of depression/anxiety predicted PPD much better than PTB. While predictive models for both PPD were improved by addition of sociodemographic factors, predictions of PPD were most improved by inclusion of mental health history and covariates related to prenatal health and pregnancy complications had the largest impact on predictions of PTB. The difference in included covariates likely reflects conceptual differences in the association between preconception mental health and a mental health outcome (PPD) and a more complex pregnancy complication. Indicators of outpatient psychiatry care and outpatient visit frequency for each period within the first five years preconception were most useful for predicting PPD and PTB, respectively. Inclusion of variables beyond a dichotomous variable for antenatal depression/anxiety significantly improved both models.

Previous studies have demonstrated a consistent link between both prenatal and preconception depression/anxiety and PPD. Using the self-reported measures of symptom severity, studies have consistently suggested that clinical and subclinical intrapartum depressive symptoms, as well as preconception diagnosis with depression are significant predictors of PPD severity [34–37]. The relationship between antenatal mental health and PTB is more ambiguous. A systematic review by Jarde et al. (2016) suggested that PTB was more likely among those with untreated and/or more severe depression, but this finding has not been consistently reported, with the complex etiology of PTB [38]. It has been further reported that the severity of prematurity is associated with perinatal depressive symptoms, indicating that a link may exist between PTB and PPD [39].

Study strengths include our use of population-based data and the 11-year period we were able to capture, allowing for more nuanced measure of exposure that is more representative of a heterogeneous depression/anxiety population. Our data suggest that use of longitudinal profiles in population-based data allow for some degree of differentiation between mild and severe cases of depression/anxiety. However, our work has some important limitations. Generalizability of our results may be limited by our requirement that birth parents be registered with MSP for over 10 years, which excluded a significant portion of the study population (84%). As indicated in Supplemental Tables 3, there were key differences between those that did and did not meet this requirement, including higher birth parent age, higher socioeconomic status (SES), and a slightly healthier profile among those with 10 years of preconception data. If we had expanded our MSP registration requirement and employed an imputation method to address missing data, we may have been able to improve generalizability of our models, particularly for those of lower SES. Birthing people of lower SES may have different profiles of mental health service use and therefore demonstrate different associations between preconception mental health history and PPD and/or PTB. This constraint also excluded recent immigrants, a population reporting higher rates of antenatal depression and PPD compared to long-term immigrants and Canadian-born individuals [29, 40]. However, we conclude that incorporating data beyond 5-years preconception did not significantly improve our model, suggesting that similar results can be realized with less restrictive data requirements in future studies.

We are also limited by missing data on therapy administered by psychologists, clinical counsellors, and/or social workers which is not covered by MSP. Our methodology further prohibits us from capturing untreated or undertreated cases of depression/anxiety, which have been differentially associated with risk for PTB [38]. Medical records potentially also underestimate symptom severity due to mismatch between mental healthcare needs and use [41]. Additionally, while we examined mental health care use over time, we did not employ a repeated measures analysis given the focus on the predictive ability of our models. We also did not distinguish between iatrogenic and spontaneous PTB, which differ mechanistically and likely weaken our predictive models.

Our results provide evidence that it is feasible to develop and apply definitions that better capture severity and longitudinal patterns of depression/anxiety when using population-based data to study perinatal mental health. Through predictive modeling for PPD and PTB, we observed better predictions and performance with inclusion of both a simple and more detailed preconception mental health history. This finding is significant as studies of perinatal mental health and psychotropic medication exposure often suffer from an inability to control for severity of underlying indication (depression/anxiety). Better adjustment for the underlying condition would allow us to clarify more ambiguous associations between antenatal mental health and adverse outcomes. By accounting for depression/anxiety severity beyond pregnancy and into preconception, we can identify cases of differential risk associated with aspects of mental health treatment, mental health history, and/or mental health in the present.

### Electronic supplementary material

Below is the link to the electronic supplementary material.

Supplementary Material 1

## Data Availability

The data supporting these findings are available from Population Data (PopData) BC (dataaccess@popdata.bc.ca) and/or directly from certain Data Steward(s)—Perinatal Services BC (psbc@phsa.ca), or the Data Innovation Program of the BC government (data@gov.bc.ca). Any researchers interested in accessing these data can apply through Population Data (PopData BC) at dataaccess@popdata.bc.ca to obtain the same dataset used in these analyses. Access to data provided by the Data Steward(s) is subject to approval, but can be requested for research projects through the Data Steward(s) or their designated service providers. All inferences, opinions, and conclusions drawn in this publication are those of the author(s), and do not reflect the opinions or policies of the Data Steward(s).

## List of Abbreviations

PPD: Postpartum depression or anxiety
PTB: Preterm birth
BC: British Columbia
PopData: Population Data
MSP: Medical Services Plan
BCPDR: BC Perinatal Data Registry
GA: Gestational age
LMP: Last menstrual period
DOC: Date of conception
DOB: Date of birth
ICD: International Classification of Disease
GDM: Gestational diabetes mellitus
PIH and non-PIH: Pregnancy-induced and non-pregnancy-induced hypertension
IUGR: Intrauterine growth restriction
NICU: Neonatal intensive care unit
SMD: Standardized difference
ROC: Receiver operating characteristic
PPV: Positive predictive value
NPV: Negative predictive value
AUROC: Area under the ROC
