# Supplementary material for: Can we more precisely classify exposure to antenatal depression and anxiety in multivariable prediction models of pregnancy and birth outcomes: a population-based cohort study
Source: BMC Psychiatry. 2023 Nov 3;23:803. doi: 10.1186/s12888-023-05284-9 (PMC10623874; doi:10.1186/s12888-023-05284-9)
Supplement: Supplementary file 1 — Supplementary Material 1 [file 12888_2023_5284_MOESM1_ESM.docx]

**Title**: Can we more precisely classify exposure to antenatal depression and anxiety in multivariable prediction models of pregnancy and birth outcomes: A population-based cohort study

**Journal name**: *BMC Psychiatry*

**Authors:** Thiele, Grace A.^1^, Ryan, Deirdre M.^2^, Oberlander, Tim F.^3^, Hanley, Gillian E.^1^

**Affiliations:**

^1^ Department of Obstetrics and Gynaecology, University of British Columbia (UBC), Vancouver, BC, 828 W 10^th^ Ave, Vancouver, BC V5Z 1M9, Canada

^2^ Departments of Psychiatry, University of British Columbia (UBC), 938 W 28^th^ Ave, Vancouver, BC V5Z 4H4, Canada

^3^ Department of Pediatrics, University of British Columbia (UBC), 938 W 28^th^ Ave, Vancouver, BC V5Z 4H4, Canada

**Corresponding author:**

Gillian E. Hanley

Vancouver General Hospital Research Pavilion

590-828 west 10^th^ Ave

Vancouver, BC Canada V5Z 1M9

Tel: 778-888-5822

[Gillian.hanley@vch.ca](mailto:Gillian.hanley@vch.ca)

Supplemental Table 1 List of included mental health conditions and corresponding diagnostic codes. MSP records primarily use the ICD-9-CM system of diagnostic codes, supplemented by a set of BC specific codes (*). DAD uses primarily ICD-10-CM diagnostic codes.

| **Diagnostic inclusion** | **Descriptor or subtype** | **MSP diagnostic code** | **DAD diagnostic code** |
| --- | --- | --- | --- |
| **Depressive diagnoses** | | | |
| Depressive disorder |  | 311.x | -- |
| Major depressive disorder | Single episode | 296.2 | F32.xx |
|  | Recurrent | 296.3 | F33.xx |
| Persistent depressive disorder | Dysthymic disorders | 300.4x | F34.1, F34.8, F34.9 |
| Psychosis | Depressive type | 298.0x | -- |
| Adjustment disorder or severe stress reaction | Adjustment disorder with depression | 309.0, 309.1 | F43.21 |
|  | Adjustment disorder with depression and anxiety | -- | F43.23 |
| Postpartum mood disturbance |  | 648.44 | F53 |
| Unspecified or other mood disorders |  | 296.9x | F38, F39 |
| **Anxiety diagnoses** | | | |
| Anxiety disorders |  | 300.0x | F41.xx |
| Adjustment disorder or severe stress reaction | Posttraumatic stress disorder | 309.81 | F43.1x |
|  | Adjustment disorder with anxiety | 309.2x | F43.22 |
| Phobic disorders | None | 300.2x | F40.xx |
| Obsessive compulsive disorders |  | 300.3x | F42.xx |
| **Other diagnoses** | | | |
| Depression or anxiety |  | 50B* | -- |

***Abbreviations***: Medical Services Plan (MSP); International Classification of Diseases, Ninth Revision, Clinical Modification (ICD-9-CM); British Columbia (BC); Discharge Abstract Database (DAD)

Supplemental Table 2 Excluded mental health conditions and corresponding diagnostic codes. MSP records primarily use the ICD-9-CM system of diagnostic codes, supplemented by a set of BC specific codes (*). DAD uses primarily ICD-10-CM diagnostic codes.

| **Diagnostic exclusion** | **Descriptor or subtype** | **MSP diagnostic code** | **DAD diagnostic code** |
| --- | --- | --- | --- |
| Schizophrenia | -- | 295.xx | F20.xx |
|  | Schizotypal disorder | 295.6 | F21.xx |
|  | Schizoaffective disorder | 295.7 | F25.xx |
| Delusional and/or psychotic disorders | -- | 297.xx | F22.xx |
|  | Brief | 293.81, 293.82, 298.3, 298.4, 298.8 | F23.xx |
|  | Shared | 297.3 | F24.xx |
|  | Other | 298.1 | F28.xx |
|  | Unspecified | 298.9 | F29.xx |
| Manic affective disorder | Recurrent episode | 296.1 | F30.xx |
| Bipolar disorders | -- | 296.xx (excluding 296.2, 296.3, and 296.9) | F31.xx |

***Abbreviations***: Medical Services Plan (MSP); International Classification of Diseases, Ninth Revision, Clinical Modification (ICD-9-CM); British Columbia (BC); Discharge Abstract Database (DAD)

**Supplemental Table 3** Comparison of sociodemographic factors, pregnancy characteristics and risk factors, and postnatal and neonatal characteristics based on inclusion status. A standardized difference of 0.1 or greater was deemed significant and designated with a (**).

|  | **Inclusion status** | | |
| --- | --- | --- | --- |
|  | **Excluded**  N = 512498 | **Included**  N = 69961 | **Standardized difference** |
| **Birth parent sociodemographic factors** | | | |
| Birth parent age group, N (%) |  |  | *0.137*** |
| < 20 years | 17173 (3.4) | 2339 (3.3) |  |
| 20 – 24 years | 71569 (14.0) | 9807 (14.0) |  |
| 24 – 29 years | 141771 (27.7) | 19639 (28.1) |  |
| 30 – 34 years | 167197 (32.6) | 22032 (31.5) |  |
| 35 – 39 years | 90983 (17.8) | 12892 (18.4) |  |
| ≥ 40 years | 19615 (3.8) | 3252 (4.6) |  |
| Missing | 4190 (0.8) | 0 (0.0) |  |
| Neighborhood income quintile, N (%) |  |  | *0.100*** |
| 1 | 114100 (23.1) | 14008 (20.0) |  |
| 2 | 107307 (21.8) | 14440 (20.6) |  |
| 3 | 99170 (20.1) | 14688 (21.0) |  |
| 4 | 93729 (19.0) | 15397 (22.0) |  |
| 5 | 79029 (16.0) | 11428 (16.3) |  |
| Marital status, N (%) |  |  | *0.215*** |
| Divorced | 8272 (1.6) | 1229 (1.8) |  |
| Married | 363864 (71.0) | 46017 (65.8) |  |
| Never married | 87636 (17.1) | 17394 (24.9) |  |
| Other | 44736 (8.7) | 4009 (5.7) |  |
| Single | 7990 (1.6) | 1312 (1.9) |  |
| Co-parents |  |  |  |
| Co-parent listed, N (%) | 488825 (95.4) | 67285 (96.2) | 0.039 |
| Co-parent age (years), Mean (SD) | 33.5 (6.4) | 33.3 (6.4) | 0.044 |
| Number of living children, N (%) |  |  | 0.033 |
| 0 | 240790 (47.0) | 32154 (46.0) |  |
| 1 | 182990 (35.7) | 25102 (35.9) |  |
| 2 | 61716 (12.0) | 8561 (12.2) |  |
| 3 | 17832 (3.5) | 2676 (3.8) |  |
| 4 or more | 9171 (1.8) | 1468 (2.1) |  |
| **Pregnancy characteristics and risk factors** | | | |
| Year of birth, Mean (SD) | 2006.1 (3.7) | 2011.9 (0.9) | *2.157*** |
| Smoked during pregnancy, N (%) |  |  | *0.156*** |
| No history of smoking | 427184 (83.4) | 56005 (80.1) |  |
| Continued during pregnancy | 51706 (10.1) | 6331 (9.0) |  |
| Discontinued during pregnancy | 33608 (6.6) | 7625 (10.9) |  |
| History of premature birth, N (%) | 20383 (4.0) | 2987 (4.3) | 0.015 |
| Nulliparous, N (%) | 236965 (46.2) | 31658 (45.3) | 0.020 |
| Diabetes, N (%) |  |  |  |
| Preexisting | 2141 (0.4) | 509 (0.7) | 0.041 |
| Gestational | 38550 (7.5) | 6494 (9.3) | 0.063 |
| Hypertension, N (%) |  |  |  |
| Pregnancy-induced | 25615 (5.0) | 3850 (5.5) | 0.023 |
| Other | 17030 (3.3) | 2484 (3.6) | 0.012 |
| Prenatal care, N (%) |  |  |  |
| ≥10 prenatal visits | 155407 (30.3) | 24596 (35.2) | *0.103*** |
| Prior hospital admissions | 52988 (10.3) | 6620 (9.5) | 0.029 |
| IUGR, N (%) | 11809 (2.3) | 1112 (1.6) | 0.052 |
| Nature of labour, N (%) |  |  |  |
| Vaginal delivery | 358203 (69.9) | 48785 (69.7) | 0.004 |
| Induced labour | 107048 (20.9) | 14628 (20.9) | 0.009 |
| Midwifery care | 46409 (9.1) | 12777 (18.3) | *0.271*** |
| Intrapartum depression or anxiety, N (%) | 93742 (18.3) | 13013 (18.6) | 0.008 |
| Depressive disorder(s) | 25218 (4.9) | 3611 (5.2) | 0.011 |
| Anxiety disorder(s) | 32586 (9.6) | 5247 (7.5) | 0.045 |
| Depression or anxiety (50B code) | 49159 (9.6) | 6164 (8.8) | 0.027 |
| **Postpartum and neonatal characteristics** | | | |
| Infant sex, N (%) | 249396 (48.7) | 34100 (48.7) | 0.002 |
| Gestational age (weeks), mean (SD) | 38.6 (2.6) | 38.6 (1.9) | 0.003 |
| Size at birth |  |  |  |
| Birth weight (g), mean (SD) | 3394.6 (579.2) | 3432.5 (547.0) | 0.067 |
| Small for gestational age, N (%) | 52440 (10.2) | 5815 (8.3) | 0.066 |
| Large for gestational age, N (%) | 50433 (9.8) | 7763 (11.1) | 0.041 |
| Admission to neonatal intensive care unit, N (%) | 10415 (2.0) | 1381 (2.0) | 0.004 |
| Preterm birth, N (%) | 48763 (9.5) | 6048 (8.6) | 0.030 |
| Postpartum depression or anxiety, N (%) | 114057 (22.3) | 15352 (21.9) | 0.008 |
| Depressive disorder(s) | 58634 (11.4) | 7664 (11.0) | 0.015 |
| Anxiety disorder(s) | 35841 (7.0) | 6063 (8.7) | 0.062 |
| Depression or anxiety (BC specific ICD-9 50B code) | 46644 (9.1) | 5771 (8.2) | 0.030 |

***Abbreviations***: standard deviation (SD), intrauterine growth restriction (IUGR), British Columbia (BC), International Classification of Diseases, Ninth Revision (ICD-9)

**Supplemental Table 4** Summary of preconception mental health characteristics among individuals with and without antenatal depression and/or anxiety. A standardized difference of 0.1 or greater was deemed significant and designated with a (**).

|  | **Intrapartum depression or anxiety** | |  |
| --- | --- | --- | --- |
|  | **No**  N = 56948 | **Yes**  N = 13013 | **Standardized difference** |
| Outpatient visit for depression or anxiety, N (%) |  |  |  |
| Between 0 – 5 yrs preconception | 23155 (40.7) | 8965 (68.9) | 0.592** |
| Between 6 – 10 yrs preconception | 19671 (34.5) | 6817 (52.4) | 0.366** |
| Prior to 10 yrs preconception | 11060 (19.4) | 3939 (30.3) | 0.253** |
| Hospitalization for depression or anxiety, N (%) |  |  |  |
| Between 0 – 5 yrs preconception | 144 (0.3) | 118 (0.9) | 0.086 |
| Between 6 – 10 yrs preconception | 237 (0.4) | 127 (1.0) | 0.067 |
| Prior to 10 yrs preconception | 54 (0.1) | 48 (0.4) | 0.057 |
| Psychiatry visit for depression or anxiety, N (%) |  |  |  |
| Between 0 – 5 yrs preconception | 1664 (2.9) | 1570 (12.1) | 0.353** |
| Between 6 – 10 yrs preconception | 1563 (2.7) | 958 (7.4) | 0.218** |
| Prior to 10 yrs preconception | 818 (1.4) | 478 (3.7) | 0.142** |
| Chronicity of depression or anxiety, N (%) |  |  | 0.619** |
| No history | 22811 (40.1) | 2434 (18.7) |  |
| Episodic (single episode) | 16195 (28.4) | 2810 (21.6) |  |
| Chronic, continuous | 15145 (26.6) | 6748 (51.9) |  |
| Chronic, discontinuous | 2797 (4.9) | 1021 (7.8) |  |

**Supplemental Table 5** Comparison of mental health characteristics across depression/anxiety diagnostic categories. A standardized difference of 0.1 or greater was deemed significant and designated with a (**).

|  | **Intrapartum depression or anxiety diagnostic categories** | | |
| --- | --- | --- | --- |
|  | **Depressive disorder(s)**  N = 3611 | **Anxiety disorder(s)**  N = 5247 | **Depression or anxiety (BC specific ICD-9 50B code)**  N = 6164 |
| Other antenatal diagnoses, N (%) |  |  |  |
| Depressive disorder(s) | -- | 817 (15.6) | 708 (11.5) |
| Anxiety disorder(s) | 817 (22.6) | -- | 667 (10.8) |
| Depression or anxiety (50B) code | 708 (19.6) | 667 (12.7) | -- |
| Outpatient visit(s) for depression or anxiety, N (%) |  |  |  |
| Between 0 – 5 yrs preconception | 3054 (84.6) | 3649 (69.5) | 4000 (64.9) |
| Between 6 – 10 yrs preconception | 2283 (63.2) | 2812 (53.6) | 3082 (50.0) |
| Prior to 10 yrs preconception | 1389 (38.5) | 1624 (31.0) | 1702 (27.6) |
| Hospitalization(s) for depression or anxiety, N (%) |  |  |  |
| Between 0 – 5 yrs preconception | 69 (1.9) | 39 (0.7) | 45 (0.7) |
| Between 6 – 10 yrs preconception | 62 (1.7) | 41 (0.8) | 59 (1.0) |
| Prior to 10 yrs preconception | 27 (0.7) | 16 (0.3) | 17 (0.3) |
| Psychiatry visit(s) for depression or anxiety, N (%) |  |  |  |
| Between 0 – 5 yrs preconception | 877 (24.3) | 686 (13.1) | 550 (8.9) |
| Between 6 – 10 yrs preconception | 468 (13.0) | 405 (7.7) | 352 (5.7) |
| Prior to 10 yrs preconception | 232 (6.4) | 190 (3.6) | 179 (2.9) |
| Chronicity of depression or anxiety, N (%) |  |  |  |
| No history | 315 (8.7) | 923 (17.6) | 1343 (21.8) |
| Episodic (single episode) | 506 (14.0) | 1148 (21.9) | 1405 (22.8) |
| Chronic, continuous | 2478 (68.6) | 2750 (52.4) | 2980 (48.3) |
| Chronic, discontinuous | 312 (8.6) | 426 (8.1) | 436 (7.1) |
| Postpartum depression or anxiety, N (%) | 2433 (67.4) | 2429 (46.3) | 2485 (40.3) |
| Depressive disorder(s) | 1926 (53.3) | 1202 (22.9) | 1076 (17.5) |
| Anxiety disorder(s) | 871 (24.1) | 1562 (29.8) | 754 (12.2) |
| Depression or anxiety (BC specific ICD-9 50B code) | 731 (20.2) | 681 (13.0) | 1565 (25.4) |

***Abbreviations:*** British Columbia (BC), International Classification of Diseases, Ninth Revision (ICD-9)

**Supplemental Table 6** Comparison of sociodemographic factors, pregnancy characteristics and risk factors, and postnatal and neonatal characteristics among individuals within the training and validation data sets. A standardized difference of 0.1 or greater was deemed significant and designated with a (**).

|  | **Predictive model building data sets** | | |
| --- | --- | --- | --- |
|  | **Test set**  N = 20988 | **Training set**  N = 48973 | **Standardized difference** |
| **Birth parent sociodemographic factors** | | | |
| Birth parent age group, N (%) |  |  | 0.006 |
| < 20 years | 716 (3.4) | 1623 (3.3) |  |
| 20 – 24 years | 2934 (14.0) | 6873 (14.0) |  |
| 24 – 29 years | 5894 (28.1) | 13745 (28.1) |  |
| 30 – 34 years | 6599 (31.4) | 15433 (31.5) |  |
| 35 – 39 years | 3866 (18.4) | 9026 (18.4) |  |
| ≥ 40 years | 979 (4.7) | 2273 (4.6) |  |
| Neighborhood income quintile, N (%) |  |  | 0.017 |
| 1 | 4198 (20.0) | 9810 (20.0) |  |
| 2 | 4379 (20.9) | 10061 (20.5) |  |
| 3 | 4326 (20.6) | 10362 (21.2) |  |
| 4 | 4603 (21.9) | 10794 (22.0) |  |
| 5 | 3482 (16.6) | 7946 (16.2) |  |
| Marital status, N (%) |  |  | 0.011 |
| Divorced | 374 (1.8) | 855 (1.7) |  |
| Married | 13824 (65.9) | 32193 (65.7) |  |
| Never married | 5176 (24.7) | 12218 (24.9) |  |
| Other | 1202 (5.7) | 2807 (5.7) |  |
| Single | 412 (2.0) | 900 (1.8) |  |
| Co-parents |  |  |  |
| Co-parent listed, N (%) | 20150 (96.0) | 47035 (96.2) | 0.018 |
| Co-parent age (years), Mean (SD) | 33.3 (6.4) | 33.3 (6.4) | 0.003 |
| Number of living children, N (%) |  |  | 0.022 |
| 0 | 9689 (46.6) | 22365 (45.7) |  |
| 1 | 7402 (35.3) | 17700 (36.1) |  |
| 2 | 2534 (12.1) | 6027 (12.3) |  |
| 3 | 820 (3.9) | 1856 (3.8) |  |
| 4 or more | 442 (2.1) | 1026 (2.1) |  |
| **Pregnancy characteristics and risk factors** | | | |
| Year of birth, Mean (SD) | 2011.9 (0.9) | 2011.9 (0.9) | 0.006 |
| Smoked during pregnancy, N (%) |  |  | 0.012 |
| No history of smoking | 16731 (79.7) | 39274 (80.2) |  |
| Continued during pregnancy | 1921 (9.2) | 4410 (9.0) |  |
| Discontinued during pregnancy | 2336 (11.1) | 5289 (10.8) |  |
| History of premature birth, N (%) | 897 (4.3) | 2090 (4.3) | <0.001 |
| Nulliparous, N (%) | 9401 (44.8) | 22257 (45.4) | 0.013 |
| Diabetes, N (%) |  |  |  |
| Preexisting | 155 (0.7) | 354 (0.7) | 0.002 |
| Gestational | 1933 (9.2) | 4561 (9.3) | 0.004 |
| Hypertension, N (%) |  |  |  |
| Pregnancy-induced | 1171 (5.6) | 2679 (5.5) | 0.005 |
| Other | 762 (3.6) | 1722 (3.5) | 0.006 |
| Prenatal care, N (%) |  |  |  |
| ≥10 prenatal visits | 7364 (35.1) | 17232 (35.2) | 0.002 |
| Prior hospital admissions | 1981 (9.4) | 4639 (9.5) | 0.001 |
| IUGR, N (%) | 350 (1.7) | 762 (1.6) | 0.009 |
| Nature of labour, N (%) |  |  |  |
| Vaginal delivery | 14572 (69.4) | 34213 (69.9) | 0.009 |
| Induced labour | 4448 (21.2) | 10180 (20.8) | 0.010 |
| Midwifery care | 3765 (17.9) | 9012 (18.4) | 0.012 |
| Intrapartum depression or anxiety, N (%) | 3898 (18.6) | 9115 (18.6) | 0.001 |
| Depressive disorder(s) | 1079 (5.1) | 2532 (5.2) | 0.001 |
| Anxiety disorder(s) | 1580 (7.5) | 3667 (7.5) | 0.002 |
| Depression or anxiety (50B code) | 1862 (8.9) | 4302 (8.8) | 0.003 |
| **Postpartum and neonatal characteristics** | | | |
| Infant sex (female), N (%) | 10153 (48.4) | 23947 (48.9) | 0.010 |
| Gestational age (weeks), mean (SD) | 38.6 (1.9) | 38.6 (1.9) | 0.003 |
| Size at birth |  |  |  |
| Birth weight (g), mean (SD) | 3432.1 (550.9) | 3432.6 (545.2) | 0.001 |
| Small for gestational age, N (%) | 1808 (8.6) | 4007 (8.2) | 0.014 |
| Large for gestational age, N (%) | 2346 (11.2) | 5417 (11.1) | 0.004 |
| Admission to neonatal intensive care unit, N (%) | 404 (1.9) | 977 (2.0) | 0.005 |
| Preterm birth, N (%) | 1809 (8.6) | 4239 (8.7) | 0.001 |
| Postpartum depression or anxiety, N (%) | 4684 (22.3) | 10668 (21.8) | 0.013 |
| Depressive disorder(s) | 2315 (11.0) | 5349 (10.9) | 0.003 |
| Anxiety disorder(s) | 1839 (8.8) | 4224 (8.6) | 0.005 |
| Depression or anxiety (BC specific ICD-9 50B code) | 1745 (8.3) | 4026 (8.2) | 0003 |

***Abbreviations***: standard deviation (SD), intrauterine growth restriction (IUGR), British Columbia (BC), International Classification of Diseases, Ninth Revision (ICD-9)

Supplemental Table 7 Comparison of logistic regression models constructed to predict the log likelihood of postpartum depression based on characterization of depression and/or anxiety history.

| **Model #** | **Time period included** | **C-statistic (95% CI)** | | **R^2^** | |
| --- | --- | --- | --- | --- | --- |
|  |  | **Binary** | **Full** | **Binary** | **Full** |
| 1 | Intrapartum (base) | 0.6268  (0.6220 – 0.6318) | 0.6341  (0.6297 – 0.6403) | 0.0953 | 0.1131 |
| 2 | **Model 1** + 0 – 1 year preconception | 0.6842  (0.6788 – 0.6896) | 0.6894  (0.6833 – 0.6955) | 0.1610 | 0.1728 |
| 3 | **Model 2** + 2 – 3 years preconception | 0.7152  (0.7096 – 0.7208) | 0.7188  (0.7130 – 0.7248) | 0.1906 | 0.2018 |
| 4 | **Model 3** + 4 – 5 years preconception | 0.7283  (0.7227 – 0.7339) | 0.7355  (0.7298 – 0.7413) | 0.2019 | 0.2124 |
| 5 | **Model 4** + 6 – 10 years preconception | 0.7349  (0.7293 – 0.7406) | 0.7389  (0.7332 – 0.7446) | 0.2068 | 0.2173 |
| 6 | **Model 5** + over 10 years preconception | 0.7363  (0.7307 – 0.7419) | 0.7398  (0.7341 – 0.7455) | 0.2080 | 0.2185 |

***Abbreviations***: coefficient of determination (R^2^), concordance statistic (C-statistic)

**Supplemental Table 8** Comparison of logistic regression models constructed to predict the log likelihood of preterm birth based on characterization of depression and/or anxiety history.

| **Model #** | **Time period included** | **C-statistic** | | **R^2^** | |
| --- | --- | --- | --- | --- | --- |
|  |  | **Binary** | **Full** | **Binary** | **Full** |
| 1 | Intrapartum (base) | 0.5123  (0.5059 – 0.5186) | 0.5139  (0.5050 – 0.5227) | 0.0007 | 0.0017 |
| 2 | **Model 1** + 0 – 1 year preconception | 0.5250  (0.5173 – 0.5327) | 0.5286  (0.5161 – 0.5344) | 0.0027 | 0.0036 |
| 3 | **Model 2** + 2 – 3 years preconception | 0.5367  (0.5281 – 0.5452) | 0.5375  (0.5345 – 0.5529) | 0.0043 | 0.0046 |
| 4 | **Model 3** + 4 – 5 years preconception | 0.5423  (0.5333 – 0.5510) | 0.5443  (0.5395 – 0.5580) | 0.0052 | 0.0060 |
| 5 | **Model 4** + 6 – 10 years preconception | 0.5440  (0.5348 – 0.5530) | 0.5457  (0.5409 – 0.5595) | 0.0053 | 0.0061 |
| 6 | **Model 5** + over 10 years preconception | 0.5451  (0.5357 – 0.5542) | 0.5470  (0.5426 – 0.5610) | 0.0058 | 0.0064 |

***Abbreviations***: coefficient of determination (R^2^), concordance statistic (C-statistic)

**Supplemental Table 9** Mean difference in fit and performance of models built using depression, anxiety, or depression or anxiety (BC specific ICD-9 50B code) indicators to predict respective postpartum depression or anxiety diagnostic categories.

| Model # | Time period included | Mean difference in C-statistic (SD) | | Mean difference in R^2^ (SD) | |
| --- | --- | --- | --- | --- | --- |
|  |  | Binary | Full | Binary | Full |
| 1 | Intrapartum (base) | 0.0073 (0.0044) | 0.0008 (0.0004) | 0.0375 (0.0277) | 0.0391 (0.0240) |
| 2 | Model 1 + 0 – 1 year preconception | 0.0195 (0.0169) | 0.0107 (0.0070) | 0.0509 (0.0372) | 0.0525 (0.0342) |
| 3 | Model 2 + 2 – 3 years preconception | 0.0263 (0.0215) | 0.0216 (0.0120) | 0.0602 (0.0451) | 0.0613 (0.0408) |
| 4 | Model 3 + 4 – 5 years preconception | 0.0269 (0.0228) | 0.0248 (0.0168) | 0.0630 (0.0465) | 0.0638 (0.0421) |
| 5 | Model 4 + 6 – 10 years preconception | 0.0308 (0.0240) | 0.0261 (0.0183) | 0.0642 (0.0482) | 0.0649 (0.0432) |
| 6 | Model 5 + over 10 years preconception | 0.0308 (0.0253) | 0.0271 (0.0189) | 0.0650 (0.0483) | 0.0658 (0.0433) |

***Abbreviations***: coefficient of determination (R^2^), standard deviation (SD), concordance statistic (C-statistic), British Columbia (BC), International Classification of Diseases, Ninth Revision (ICD-9)

**Supplemental Table 10** Mean difference in fit and performance of models built using depression, anxiety, or depression or anxiety (BC specific ICD-9 50B code) indicators to predict preterm birth.

| Model # | Time period included | Mean difference in C-statistic | | Mean difference in R^2^ | |
| --- | --- | --- | --- | --- | --- |
|  |  | Binary | Full | Binary | Full |
| 1 | Intrapartum (base) | 0.0036 (0.0026) | 0.0009 (0.0005) | 0.0004 (0.0002) | 0.0001 (0.0001) |
| 2 | Model 1 + 0 – 1 year preconception | 0.0090 (0.0039) | 0.0019 (0.0013) | 0.0011 (0.0007) | 0.0004 (0.0003) |
| 3 | Model 2 + 2 – 3 years preconception | 0.0035 (0.0025) | 0.0022 (0.0012) | 0.0007 (0.0004) | 0.0002 (0.0002) |
| 4 | Model 3 + 4 – 5 years preconception | 0.0041 (0.0030) | 0.0010 (0.0004) | 0.0009 (0.0006) | 0.0001 (0.0001) |
| 5 | Model 4 + 6 – 10 years preconception | 0.0069 (0.0042) | 0.0013 (0.0006) | 0.0012 (0.0007) | 0.0002 (0.0001) |
| 6 | Model 5 + over 10 years preconception | 0.0062 (0.0028) | 0.0021 (0.0009) | 0.0013 (0.0006) | 0.0005 (0.0003) |

***Abbreviations***: coefficient of determination (R^2^), standard deviation (SD), concordance statistic (C-statistic), British Columbia (BC), International Classification of Diseases, Ninth Revision (ICD-9)

Supplemental Table 11 Summary of final adjusted model for predicting likelihood postpartum depression and/or anxiety (PPD).

| **Performance measures** | | |
| --- | --- | --- |
| Sensitivity (95% CI) | 0.6078 (0.5937 – 0.6218) | |
| Specificity (95% CI) | 0.7559 (0.7492 – 0.7492) | |
| PPV (95% CI) | 0.4170 (0.4053 – 0.4288) | |
| NPV (95% CI) | 0.8703 (0.8646 – 0.8758) | |
| **Equation:** $Probability of PPD\boldsymbol{=}\frac{\boldsymbol{1}}{\boldsymbol{1+}\boldsymbol{e}^{\boldsymbol{-(}\boldsymbol{b}_{\boldsymbol{0}}\boldsymbol{+}\boldsymbol{b}_{\boldsymbol{1}}\boldsymbol{x}_{\boldsymbol{1}}\boldsymbol{+\ldots+}\boldsymbol{b}_{\boldsymbol{n}}\boldsymbol{x}_{\boldsymbol{n}}\boldsymbol{)}}}$  **Directions**: Corresponding coefficients (b) and input values (x) listed below can be put into the formula above to determine the estimate probability of developing postpartum depression and/or anxiety (PPD). | | |
| **Covariate** | **Coefficient (b)** | **Input value (x)** |
| Intercept | -1.8815 |  |
| Depression or anxiety (pregnancy) | 0.8158 | Yes = 1 |
| Number of outpatient visits (pregnancy) | 0 | 0 visits = 1 |
|  | -0.0073 | 1 visit = 1 |
|  | -0.0305 | 2 or more visits = 1 |
| Hospitalization (pregnancy) | 0.7664 | Yes = 1 |
| Psychiatry visit (pregnancy) | 1.3833 | Yes = 1 |
| Depression or anxiety  (0 – 1 year preconception) | 0.7784 | Yes = 1 |
| Psychiatry visit  (0 – 1 year preconception) | 0.2149 | Yes = 1 |
| Depression or anxiety  (2 – 3 years preconception) | 0.4912 | Yes = 1 |
| Psychiatry visit  (2 – 3 years preconception) | 0.3648 | Yes = 1 |
| Depression or anxiety  (4 – 5 years preconception) | 0.3625 | Yes = 1 |
| Psychiatry visit  (4 – 5 years preconception) | 0.1264 | Yes = 1 |
| Chronicity | 0 | No prior history = 1 |
|  | 0.4837 | Continuous = 1 |
|  | 0.3489 | Discontinuous = 1 |
|  | 0.2227 | Episodic = 1 |
| Birth parent age | 0 | < 20 years = 1 |
|  | -0.1543 | 20 – 24 years = 1 |
|  | -0.2821 | 25 – 29 years = 1 |
|  | -0.3479 | 30 – 34 years = 1 |
|  | -0.3351 | 35 – 39 years = 1 |
|  | -0.3761 | > 40 years = 1 |
| Marital status | 0 | Divorced = 1 |
|  | -0.1155 | Married = 1 |
|  | -0.0081 | Never married = 1 |
|  | 0.0756 | Other = 1 |
|  | 0.0509 | Single = 1 |
| Smoking history | 0 | No history = 1 |
|  | 0.1878 | Continued during pregnancy = 1 |
|  | 0.2006 | Discontinued during pregnancy = 1 |
| Number of living children | 0 | 0 children = 1 |
|  | -0.1120 | 1 child = 1 |
|  | -0.1508 | 2 children = 1 |
|  | -0.0561 | 3 children = 1 |
|  | -0.2572 | 4 or more children = 1 |
| Midwifery care during labor | -0.2748 | Yes = 1 |
| Antenatal hospitalization | 0.1547 | Yes = 1 |
| Admission to NICU | 0.1788 | Yes = 1 |
| > 10 prenatal visits | 0.0787 | Yes = 1 |
| Premature birth | 0.1146 | Yes = 1 |

***Abbreviations:*** confidence interval (CI), positive predictive value (PPV), negative predictive value (NPV), neonatal intensive care unit (NICU)

Supplemental Table 12 Summary of final adjusted model for predicting likelihood of preterm birth (PTB).

| **Performance measures** | | |
| --- | --- | --- |
| Sensitivity (95% CI) | 0.6611 (0.6388 – 0.6830) | |
| Specificity (95% CI) | 0.6683 (0.6616 – 0.6750) | |
| PPV (95% CI) | 0.1583 (0.1501 – 0.1667) | |
| NPV (95% CI) | 0.9544 (0.9507 – 0.9578) | |
| **Equation:** $Probability of PTB\boldsymbol{=}\frac{\boldsymbol{1}}{\boldsymbol{1+}\boldsymbol{e}^{\boldsymbol{-(}\boldsymbol{b}_{\boldsymbol{0}}\boldsymbol{+}\boldsymbol{b}_{\boldsymbol{1}}\boldsymbol{x}_{\boldsymbol{1}}\boldsymbol{+\ldots+}\boldsymbol{b}_{\boldsymbol{n}}\boldsymbol{x}_{\boldsymbol{n}}\boldsymbol{)}}}$  **Directions**: Corresponding coefficients (b) and input values (x) listed below can be put into the formula above to determine the estimate probability of developing preterm birth (PTB). | | |
| **Covariate** | **Coefficient (b)** | **Input value (x)** |
| Intercept | -2.3585 |  |
| Depression or anxiety (pregnancy) | -0.0664 | Yes = 1 |
| Number of outpatient visits (pregnancy) | 0 | 0 visits = 1 |
|  | 0.1143 | 1 visit = 1 |
|  | 0.0645 | 2 or more visits = 1 |
| Hospitalization (pregnancy) | 0.5014 | Yes = 1 |
| Psychiatry visit (pregnancy) | 0.2665 | Yes = 1 |
| Depression or anxiety  (0 – 1 year preconception) | 0.1139 | Yes = 1 |
| Number of outpatient visits  (0 – 1 year preconception) | 0 | 0 visits = 1 |
|  | -0.0887 | 1 visit = 1 |
|  | -0.0728 | 2 or more visits = 1 |
| Depression or anxiety  (2 – 3 years preconception) | -0.0018 | Yes = 1 |
| Number of outpatient visits  (2 – 3 years preconception) | 0 | 0 visits = 1 |
|  | 0.0845 | 1 visit = 1 |
|  | 0.1790 | 2 or more visits = 1 |
| Depression or anxiety  (4 – 5 years preconception) | 0.0600 | Yes = 1 |
| Number of outpatient visits  (4 – 5 years preconception) | 0 | 0 visits = 1 |
|  | -0.0907 | 1 visit = 1 |
|  | 0.0206 | 2 or more visits = 1 |
| Chronicity | 0 | No prior history |
|  | 0.0206 | Continuous = 1 |
|  | 0.0665 | Discontinuous = 1 |
|  | -0.0278 | Episodic = 1 |
| Birth parent age | 0 | < 20 years = 1 |
|  | -0.0036 | 20 – 24 years = 1 |
|  | 0.1002 | 25 – 29 years = 1 |
|  | 0.0510 | 30 – 34 years = 1 |
|  | 0.1782 | 35 – 39 years = 1 |
|  | 0.2286 | > 40 years = 1 |
| Marital status | 0 | Divorced |
|  | -0.1745 | Married = 1 |
|  | -0.1169 | Never married = 1 |
|  | 0.1112 | Other = 1 |
|  | 0.0939 | Single = 1 |
| Smoking history | 0 | No history = 1 |
|  | 0.2075 | Continued during pregnancy = 1 |
|  | 0.0037 | Discontinued during pregnancy = 1 |
| Number of living children | 0 | 0 children = 1 |
|  | -0.3287 | 1 child = 1 |
|  | -0.2738 | 2 children = 1 |
|  | -0.1674 | 3 children = 1 |
|  | -0.1014 | 4 or more children = 1 |
| History of premature birth | 1.4517 | Yes = 1 |
| Pre-existing diabetes | 1.1511 | Yes = 1 |
| Gestational diabetes | 0.4159 | Yes = 1 |
| PIH | 0.4839 | Yes = 1 |
| Non-PIH | 0.7177 | Yes = 1 |
| IUGR | 1.4775 | Yes = 1 |
| Midwifery care during labor | -0.2920 | Yes = 1 |
| Antenatal hospitalization | 1.1346 | Yes = 1 |
| Infant sex | 0.1029 | Non-female = 1 |
| > 10 prenatal visits | -1.1810 | Yes = 1 |

***Abbreviations:*** confidence interval (CI), positive predictive value (PPV), negative predictive value (NPV), pregnancy induced hypertension (PIH), intrauterine growth restriction (IUGR), neonatal intensive care unit (NICU)
